# Empowering Healthy Habits Through Community Based Hypertension Screening and Counseling by Healthcare Students

**DOI:** 10.1093/geroni/igaf122.3923

**Published:** 2025-12-31

**Authors:** Don’aa Williams, Taqwa Naas, Anna Azuz, Maria Sobhie, Jennifer Mendez, Brittany Stewart, Rima Charara

**Affiliations:** Wayne State University School of Medicine, Detroit, Michigan, United States; Wayne State University, Detroit, Michigan, United States; Wayne State University, Detroit, Michigan, United States; Wayne State University, Detroit, Michigan, United States; Wayne State University School of Medicine, Detroit, Michigan, United States; Wayne State University, Detroit, Michigan, United States; Wayne State University School of Medicine, Detroit, Michigan, United States

## Abstract

Hypertension is highly prevalent in the community, yet it often goes undetected, especially among older adults with limited access to care. Although blood pressure is routinely checked during doctor visits, individuals who do not seek regular care may dismiss their symptoms. As a chronic, multifactorial illness, hypertension can lead to poor health outcomes and is known as a “silent killer.” To address this, Wayne State healthcare students provide free blood pressure screenings and health counseling to the community every week from June to August at the Detroit Riverwalk. Supervised by physicians and pharmacists, students gain hands-on experience conducting holistic assessments and providing medication and lifestyle counseling, with particular attention to hypertension among older adults. In the summer of 2025 alone, the program conducted approximately 470 screenings. A total of 82 students participated, including 66 medical students and 16 pharmacy students, all of whom completed the AMA blood pressure training modules prior to volunteering. These in-person screenings not only strengthened students’ skills in accurate blood pressure measurement but also provided valuable experience in patient-centered counseling and interprofessional collaboration. Riverwalk participants who agreed to be screened received health resources, information on free clinics and primary care providers, as well as nutrition guidance. Survey data revealed that participants left screenings with greater confidence in managing their blood pressure and a strong intention to continue engaging in future health checks. This initiative demonstrates a replicable model of community-centered, interprofessional education that enhances student learning while building trust and promoting autonomy among older adults.

